# ICTV Virus Taxonomy Profile: *Corticoviridae*

**DOI:** 10.1099/jgv.0.000795

**Published:** 2017-06-05

**Authors:** Hanna M. Oksanen, ICTV Report Consortium

**Affiliations:** Department of Biosciences and Institute of Biotechnology, University of Helsinki, FIN-00014 Helsinki, Finland

**Keywords:** ICTV Report, taxonomy, *Corticoviridae*

## Abstract

The *Corticoviridae* is a family of icosahedral, internal-membrane-containing viruses with double-stranded circular DNA genomes of approximately 10 kb. Only one species, *Pseudoalteromonas virus PM2*, has been recognized. Pseudoalteromonas virus PM2 infects Gram-negative bacteria and was isolated from seawater in 1968. Pseudoalteromonas virus PM2 is the first bacterial virus in which the presence of lipids in the virion has been demonstrated. Viral lipids are acquired selectively during virion assembly from the host cytoplasmic membrane. The outer protein capsid is an icosahedron with a pseudo *T*=21 symmetry. This is a summary of the International Committee on Taxonomy of Viruses (ICTV) Report on the taxonomy of the *Corticoviridae*, which is available at www.ictv.global/report/corticoviridae.

## Virion

**Fig. 1.**
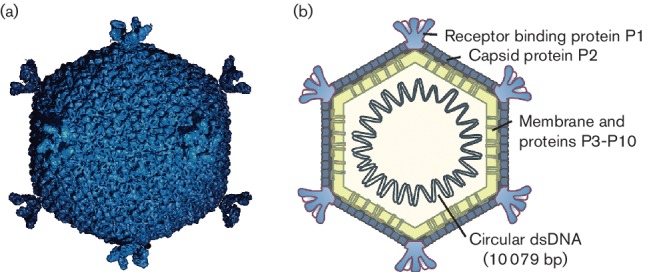
(a) An atomic model based upon X-ray crystallographic analysis of a virion of Pseudoalteromonas virus PM2 at 7 Å resolution, viewed along the two fold axis of symmetry (courtesy of N.G.A. Abrescia) and (b) a schematic presentation of the virion. Capsid diameter is approximately 57 nm (from facet to facet).

Icosahedral virions consist of an internal membrane and an outer protein capsid that has a diameter of 57 nm between facets (Table 1, Fig. 1). The genome is enclosed by the membrane. The capsid consists of 200 major capsid protein P2 trimers that are organized on a pseudo *T*=21 lattice [1]. Spikes protruding about 8 nm from the capsid surface at the fivefold vertices are homopentamers and are formed of protein P1. The viral lipids (mainly phosphatidyl ethanolamine and phosphatidyl glycerol) are derived from the host plasma membrane, but their composition deviates from that of the host bacterium [2–4]. Lipids form an internal membrane with virus-specific membrane-associated proteins.

**Table 1.** Characteristics of the family *Corticoviridae*

Typical member:	Pseudoalteromonas virus PM2 (AF155037), species *Pseudoalteromonas virus PM2*, genus *Corticovirus*
Virion	Icosahedral, internal-membrane-containing virions of about 57 nm with a single capsid protein P2, a single spike protein P1 and eight membrane-associated proteins P3–P10
Genome	10.1 kb of highly supercoiled circular double-stranded DNA
Replication	Rolling circle replication initiated by virus-encoded protein P12
Translation	Prokaryotic translation using viral mRNA and host ribosomes
Host range	Bacteria, Gram-negative *Pseudoalteromonas* strains
Taxonomy	One genus containing one species

## Genome

The genome is a highly supercoiled, circular, double-stranded DNA of 10 079 bp [5]. The DNA comprises about 14 % of the virion weight, and the G+C content is 42.2 %. The genome has 21 putative genes, 10 of which have been shown to code for structural proteins (P1–P10), seven of which encode nonstructural proteins (P12–P18) and four of which are of unknown function.

## Replication

**Fig. 2.**
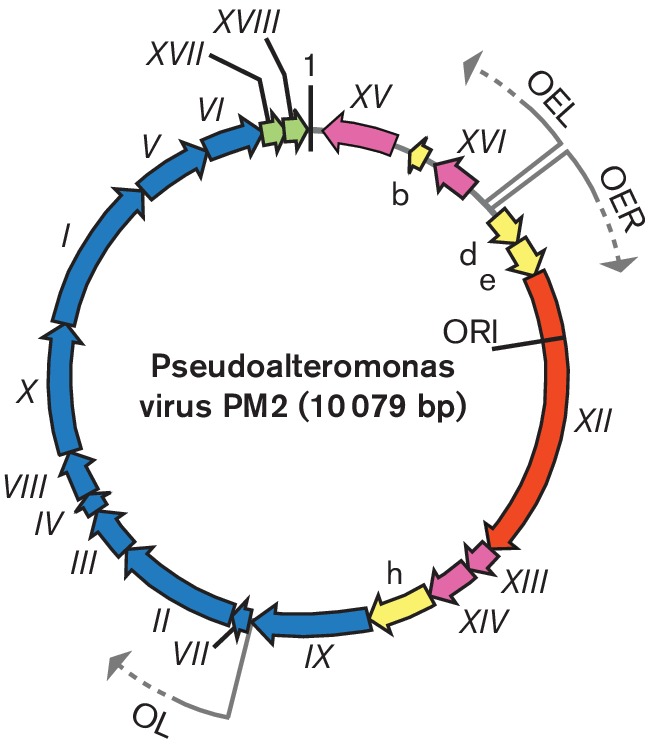
Genome organization of Pseudoalteromonas virus PM2. The genome is a 10 079 bp, highly supercoiled, circular, double-stranded DNA molecule containing 17 genes (Roman numerals) and four additional ORFs (letters). The arrows indicate the orientations and three operons (OER, OEL and OL). ORFs known to code for functional proteins are classified as genes and are given a Roman numeral. The different colours indicate the ORFs encoding putative proteins (yellow), a gene for replication initiation protein (orange) and the following groups of genes: transcriptional regulation (magenta), structural proteins (blue) and lysis (green). Positions of the origin of replication (ORI) and the first nucleotide (marked as 1) are indicated.

Replication of the Pseudoalteromonas virus PM2 genome, most probably by a rolling circle mechanism, takes place in proximity to the host cytoplasmic membrane. The largest virus protein P12, encoded by gene *XII*, shares significant sequence similarity with the superfamily I group of replication initiation proteins [5]. The genome is organized into three operons (Fig. 2). Operons OEL and OER encode early gene products: the replication initiation protein P12 and transcriptional regulatory proteins. Expression of the genes for structural proteins is under the control of the late promoter (OL), which is activated by the virus-encoded transcription factors.

## Taxonomy

The *Corticoviridae* family consists of only the genus *Corticovirus*, containing one recognized species – *Pseudoalteromonas virus PM2* (Table 1). The only isolate, Pseudoalteromonas virus PM2, is a virulent virus infecting Gram-negative *Pseudoalteromonas* species [6, 7].

## Resources

Full ICTV Online (10th) Report: www.ictv.global/report/corticoviridae.

## References

[1] Abrescia NG, Grimes JM, Kivelä HM, Assenberg R, Sutton GC (2008). Insights into virus evolution and membrane biogenesis from the structure of the marine lipid-containing bacteriophage PM2. Mol Cell.

[2] Braunstein SN, Franklin RM (1971). Structure and synthesis of a lipid-containing bacteriophage. V. Phospholipids of the host BAL-31 and of the bacteriophage PM2. Virology.

[3] Camerini-Otero RD, Franklin RM (1972). Structure and synthesis of a lipid-containing bacteriophage. XII. The fatty acids and lipid content of bacteriophage PM2. Virology.

[4] Kivelä HM, Kalkkinen N, Bamford DH (2002). Bacteriophage PM2 has a protein capsid surrounding a spherical proteinaceous lipid core. J Virol.

[5] Männistö RH, Kivelä HM, Paulin L, Bamford DH, Bamford JK (1999). The complete genome sequence of PM2, the first lipid-containing bacterial virus to be isolated. Virology.

[6] Espejo RT, Canelo ES (1968). Properties of bacteriophage PM2: a lipid-containing bacterial virus. Virology.

[7] Kivelä HM, Männistö RH, Kalkkinen N, Bamford DH (1999). Purification and protein composition of PM2, the first lipid-containing bacterial virus to be isolated. Virology.

